# Improvement of survival in Korean breast cancer patients over a 14-year period: A large-scale single-center study

**DOI:** 10.1371/journal.pone.0265533

**Published:** 2022-03-16

**Authors:** Hakyoung Kim, Sae Byul Lee, Jisun Kim, Il Yong Chung, Hee Jeong Kim, Beom Seok Ko, Jong Won Lee, Byung Ho Son, Sei Hyun Ahn

**Affiliations:** Division of Breast Surgery, Department of Surgery, University of Ulsan College of Medicine, Asan Medical Center, Seoul, Republic of Korea; University Magna Graecia of Catanzaro, ITALY

## Abstract

**Purpose:**

The aim of this study was to evaluate the chronological changes over 14 years in the survival of Korean patients with breast cancer. We also sought to investigate the factors that may have influenced the changes in survival rate.

**Materials and methods:**

We retrospectively analyzed 17,776 breast cancer patients who were treated at Asan Medical Center between January 2000 and December 2013. Patient information was collected from the Asan database, including age at diagnosis, clinical manifestation, pathology report, types of treatment and modality, types of recurrence, and follow-up period. We classified the patients into two cohorts according to the year of their surgery (P1: 2000–2007 and P2: 2008–2013) and compared survival and recurrence between both cohorts.

**Results:**

We observed that patients treated more recently had better survival outcomes. The 5-year breast cancer-specific survival increased from 94.0% in P1 to 96.6% in P2 (p<0.001), and the 5-year disease-free survival increased from 87.9% in P1 to 91.2% in P2 (p<0.001). When analyzed by type of recurrence, distant metastasis-free survival increased to a significant degree. In subgroup analysis by the subtypes of breast cancer, the survival rates improved in all of the subtypes except triple negative breast cancer, and the improvement was more prominent in subtypes with overexpressed human epidermal growth factor receptor 2.

**Conclusion:**

This study showed improvement in breast cancer survival over the succeeding years, which is consistent with the advancement in systemic therapy.

## Introduction

Breast cancer is the most commonly diagnosed cancer and the leading cause of cancer death among females worldwide, accounting for 24.2% of total cancer incidence and 15% of total cancer mortality [1]. There is substantial diversity in cancer incidence and death among different countries, with Korea being one of the countries with the lowest breast cancer incidence rates (50.7 per 100,000 women per year in 2012) [2–5]. However, the incidence has risen rapidly and, according to the Korean National Cancer Center data, breast cancer is now the most common cancer among Korean women, accounting for 23.8% of total female cancer incidence [6–8].

Recently, many studies in western countries have reported improvement in the survival of breast cancer patients, and provided a few possible explanations for this trend [9]. These include nationwide screening programs that lead to early detection of breast cancer [10], an increased proportion of less aggressive types of breast cancer [9], and advancement in adjuvant therapy [11–14]. According to statistics from the national breast cancer data obtained from the Korean Breast Cancer Registry, survival of Korean breast cancer has also improved similar to many other countries. Five-year relative survival for breast cancer continuously increased from 79.2% in patients diagnosed between 1993 to 1995, to 88.7% in patients diagnosed between year 2001 to 2005 [4, 9, 15]. Korea has nationwide screening program providing mammography every 2 year to women over 40 years old since 1999 and this may be one of the reasons for improvement in survival. However, unlike western countries, there are few investigations that have analyzed underlying causes for improvement in the survival of Korean patients.

Aim of this study was to evaluate the changing patterns of survival and recurrence in Korean breast cancer patients over a 14-year period (2000–2013) and analyze the factors that may have influenced the changes in survival rate.

## Materials and methods

Asan medical center has one of the biggest breast cancer clinic in Korea and more than 2,000 breast cancer patients under go operation in this hospital each year, which is about 10% of total breast cancer patients in Korea. In this retrospective single-center study, we used the Asan database, which was a prospectively collected database of breast cancer patients treated at Asan Medical Center that provided information on age, clinical manifestations, pathology reports, types and modality of treatment, types of recurrence, and follow-up period.

### Patient and study design

Patients who were diagnosed with breast cancer at Asan medical center between January 2000 and December 2013 were enrolled in this study. Total of 18,185 patients were reviewed and stage IV breast cancer patients, patients who did not undergo operation, patient with 0 follow-up period and patients with other malignant breast diseases, such as phyllodes tumors, lymphoma, or sarcoma were excluded. Ultimately, 17,776 patients who were diagnosed with stage 0–3 breast cancer patients and underwent surgery at Asan Medical Center were included.

We divided the patients into two groups, based on the year of their operation: P1 = 2000–2007 and P2 = 2008–2013.The overall survival (OS), breast cancer-specific survival (BCSS), and disease-free survival (DFS) were compared between the two groups. Follow up was censored at the earliest of these three; death, last hospital follow up or 31^st^ of December, 2018.

Because we included patients who underwent neoadjuvant chemotherapy, we used pathological TNM staging for those who underwent upfront surgery, whereas clinical TNM staging for those who underwent neoadjuvant chemotherapy. The TNM stage was assigned according to the American Joint Committee on Cancer 7^th^ classification.

To evaluate the factors that affected the chronological survival difference, we collected information on each patient’s adjuvant treatment. Treatment varied depending on each patient’s general condition, tumor stage, and tumor subtype. The types of hormone treatment used were aromatase inhibitor (AI), selective estrogen receptor modulator (SERM), and luteinizing hormone-releasing hormone (LHRH) analog. Adjuvant chemotherapy was categorized into four types: anthracycline-based, anthracycline and taxane-based, cyclophosphamide, methotrexate, and 5-fluorouracil (CMF), or others. Starting from 2007, the use of trastuzumab as an adjuvant treatment for advanced breast cancer with human epidermal growth factor receptor 2 (HER2) overexpression was covered by the Korean National Health Insurance; since then, the use of trastuzumab has increased in Korea.

We performed a subgroup analysis of the OS, BCSS, and DFS by breast cancer subtype. The subtype was designated depending on the status of hormone receptors and overexpression of HER2 receptors: luminal A (hormone receptor [+] and HER2 [−]), luminal B (hormone receptor [+] and HER2 [+]), HER2 type (hormone receptor [−] and HER2 [+]), and triple negative (hormone receptor [−] and HER2 [−]).

### Pathological data

Pathological data were evaluated at the Department of Pathology at the Asan Medical Center. Immunohistochemistry (IHC) was used to determine estrogen receptor status, progesterone receptor status, and HER2 status. The estrogen receptor and progesterone receptor statuses were considered positive if >10% of cells were positive. For HER2 overexpression, patients with IHC grade 0, 1+ were considered to be negative, and patients with IHC grade 3+ were considered to be positive. Cases of IHC grade 2+were further evaluated by fluorescence *in situ* hybridization.

### Statistical analysis

A chi-square test was used to compare clinicopathological parameters of two period of time. OS was defined as the time from the initial surgery to the time of death, BCSS was defined as the time from the initial surgery to the time of death caused by breast cancer, and DFS was defined as the time from the initial surgery to the date of the first appearance of relapse, regardless of local, regional, or systemic recurrence. The Kaplan–Meier method was used to estimate survival curves, and the significance of survival differences among selected variables was verified using the log-rank test. Cox proportional hazards models were used for univariable and multivariable analysis. All reported p-values were two-sided, and a value of p<0.05 was considered statistically significant. We conducted our analysis using the SPSS statistical software version 21 (SPSS Inc., Chicago, USA) and R4.1.1.

### Ethical approval

All of the procedures performed in the study were in accordance with the ethical standards of the institutional and/or national research committee and with the 1964 Helsinki Declaration and its later amendments or comparable ethical standards.

This study was approved by the Asan Medical Center review board (IRB No. 2018–0079). The requirement of informed consent was waived because the study was based on retrospective clinical data

## Results

### Patient characteristics

A total of 17,776 patients were included in this study. Of these, 7,066 patients underwent surgery in 2000–2007 (P1), while 10,710 patients underwent surgery in 2008–2013 (P2). The clinicopathological features are shown in Table 1.

**Table 1:** Clinicopathological characteristics of patients according to the period at diagnosis.

Factors	2000–2007	2008–2013	Total	p-value
	(n = 7,066)	(n = 10,710)	(n = 17,776)	
	No. (%)	No. (%)	No. (%)	
Age at diagnosis (y)				<0.001
<50	4,509 (63.8)	5,913 (55.2)	10,422 (58.6)	
≥50	2,557 (36.2)	4,797 (44.8)	7,354 (41.4)	
T stage				<0.001
Tis	658 (9.3)	1,175 (11.0)	1,833 (10.3)	
T1	3,531 (50.0)	5,432 (50.7)	8,963 (50.4)	
T2	2,517 (35.6)	3,444 (32.2)	5,961 (33.5)	
T3	259 (3.7)	503 (4.7)	762 (4.3)	
T4	99 (1.4)	108 (1.0)	207 (1.2)	
T0	1 (0.0)	7 (0.1)	8 (0.0)	
Unknown	1 (0.0)	41 (0.4)	42 (0.2)	
N stage				<0.001
N0	4,499 (63.7)	7,177 (67.0)	11,676 (65.7)	
N1	1,782 (25.2)	2,507 (23.4)	4,289 (24.1)	
N2	463 (6.6)	539 (5.0)	1,002 (5.6)	
N3	322 (4.6)	444 (4.1)	766 (4.3)	
Unknown	0(0.0)	43 (0.4)	43(0.2)	
Histologic grade				<0.001
G1	414 (5.9)	571 (5.3)	985 (5.5)	
G2	3,178 (45.0)	5,346 (49.9)	8,524 (48.0)	
G3	2,211 (31.3)	3,282 (30.6)	5,493 (30.9)	
Unknown	1,263 (17.9)	1,511 (14.1)	2,774 (15.6)	
Nuclear grade				<0.001
G1	367 (5.2)	730 (6.8)	1,097 (6.2)	
G2	3,037 (43.0)	5,980 (55.8)	9,017 (50.7)	
G3	2,067 (29.3)	3,572 (33.4)	5,639 (31.7)	
Unknown	1,595 (22.6)	428 (4.0)	2,023 (11.4)	
Lymphovascular invasion				<0.001
Negative	3,968 (56.2)	7,060 (65.9)	11,028 (62.0)	
Positive	1,425 (20.2)	2,126 (19.9)	3,551 (20.0)	
Unknown	1,673 (23.7)	1,524 (14.2)	3,197 (18.0)	
Hormone receptor				<0.001
Yes	4,643 (65.7)	7,421 (69.3)	12,064 (67.9)	
No	2,252 (31.9)	3,057 (28.5)	5,309 (29.9)	
Unknown	171 (2.4)	232 (2.2)	403 (2.3)	
HER-2				<0.001
Negative	4,897 (69.3)	8,067 (75.3)	12,964 (72.9)	
Positive	1,872 (26.5)	2,389 (22.3)	4,261 (24.0)	
Unknown	297 (4.2)	254 (2.4)	551 (3.1)	
Subtype				<0.001
HR+/HER2−	3,633 (51.4)	6,366 (59.4)	9,999 (56.3)	
HR+/HER2+	930 (13.2)	1,031 (9.6)	1,961 (11.0)	
HR−/HER2+	941 (13.3)	1,356 (12.7)	2,297 (12.9)	
HR−/HER2−	1,264 (17.9)	1,700 (15.9)	2,964 (16.7)	
Unknown	298 (4.2)	257 (2.4)	555 (3.1)	
Breast operation				<0.001
Breast-conserving surgery	3,159 (44.7)	7,143 (66.7)	10,302 (58.0)	
Total mastectomy	3,907 (55.3)	3,567 (33.3)	7,474 (42.0)	
Axillary operation				<0.001
SNB	980 (13.9)	6,612 (61.7)	7,592 (42.7)	
ALND	5,718 (80.9)	3,396 (31.7)	9,114 (51.3)	
No operation	368 (5.2)	702 (6.6)	1,070 (6.0)	
Neoadjuvant therapy				<0.001
Yes	6 (0.1)	1,309 (12.2)	1,315 (7.4)	
No	7,060 (99.9)	9,401 (87.8)	16,461 (92.6)	
Chemotherapy				<0.001
Yes	4,535 (64.2)	5,890 (55.0)	10,425 (58.6)	
No	2,476 (35.0)	4,765 (44.5)	7,241 (40.7)	
Unknown	55 (0.8)	55 (0.5)	110 (0.6)	
Hormonal therapy				<0.001
Yes	4,687 (66.3)	7,367 (68.8)	12,054 (67.8)	
No	2,293 (32.5)	3,249 (30.3)	5,542 (31.2)	
Unknown	86 (1.2)	94 (0.9)	180 (1.0)	
Target therapy				<0.001
Yes	34 (0.5)	1,505 (14.1)	1,539 (8.7)	
No	7,031(99.5)	9,068 (84.7)	14,097 (79.3)	
Unknown	0 (0.0)	137 (1.3)	2,140 (12.0)	
Radio therapy				<0.001
Yes	3,948 (55.9)	7,802 (72.8)	11,750 (66.1)	
No	3,083 (43.6)	2,840 (26.5)	5,923 (33.3)	
Unknown	35 (0.5)	68 (0.6)	103 (0.6)	

Stage: initial clinical stage for patients who underwent neoadjuvant therapy and pathological stage for patients who did not; ALND: axillary lymph node dissection; HER2: human epidermal growth factor receptor 2; HR: hormone receptor; HR+: estrogen receptor positive or progesterone receptor positive; SNB: sentinel lymph node biopsy.

While more than 50% of the patients were diagnosed with breast cancer before the age of 50 throughout the whole study period, the proportion of older patients increased from P1 to P2 (36.2% in P1 to 44.8% in P2, p<0.001). In addition, the proportion of patients who were diagnosed during the early stages of breast cancer increased slightly over time. As for the type of breast and axillary surgery, the proportion of patients who underwent breast-conserving surgery and/or sentinel lymph node biopsy increased markedly over the study period. Patients who received neoadjuvant therapy also increased, with 12.2% of patients receiving neoadjuvant therapy in P2. Patients who received HER2-targeted therapy also increased from 0.5% in P1 to 14.1% in P2 (p<0.001).

### Analysis according to the period

**Fig 1:**
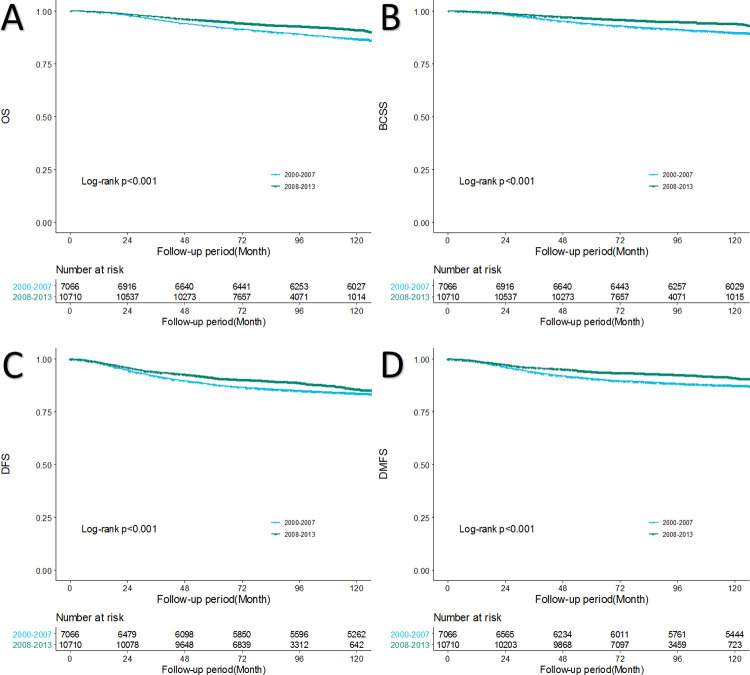
Chronological changes of survival in Korean breast cancer patients. (A) Overall survival (OS). (B) Breast cancer-specific survival (BCSS). (C) Disease-free survival (DFS). (D) Distant metastasis-free survival (DMFS).

In the Kaplan–Meier survival curve, the OS, BCSS, DFS and distant metastasis-free survival (DMFS) of the P2 cohort were significantly better than those of the P1 cohort (**Fig 1**). The 5-year OS increased from 92.6% in P1 to 95.3% in P2 and the 5-year BCSS increased from 94.0% in P1 to 96.6% in P2. DFS especially improved throughout the study period, with the 5-year DFS increasing from 87.9% in P1 to 91.2% in P2. The 5-year DMFS was 87.9% in P1 and 91.2% in P2. The median follow-up was 155.9 months for P1 and 85.9 months for P2.

We performed univariate and multivariate Cox proportional hazards regression analysis for OS and DFS. Variants included in the analysis were time of surgery, age, T-stage, N-stage, histological grade, nuclear grade, lymphovascular invasion status, hormone receptor status, HER2 overexpression status, hormonal therapy status, chemotherapy status, and HER2-targeted therapy status. The analysis demonstrated that the time factor (P1 or P2) itself was independently and significantly associated with OS, DFS (Table 2). In multivariate analysis, P2 exhibited better OS and DFS with hazard ratios of 0.773 (0.684–0.873, p <0.001) and 0.875(0.788–0.972, p = 0.013), respectively.

**Table 2:** Univariate and multivariate Cox regression analysis for OS, and DFS according to the period of surgery.

	Univariate		Multivariate	
	p-value	Hazard ratio (95% CI)	p-value	Hazard ratio (95% CI)
OS				
Year of surgery (2000–2007 vs 2008–2013)	<0.001	0.646(0.585–0.714)	0.001	0.773(0.684–0.873)
Age (<50 vs ≥50)	<0.001	1.337(1.222–1.463)	<0.001	1.318(1.190–1.460)
Histologic grade (G1 vs)	<0.001		0.073	
G2	<0.001	2.715(1.935–3.810)	0.036	1.676(1.034–2.717)
G3	<0.001	5.156(3.679–7.227)	0.016	1.875(1.122–3.134)
Nuclear grade (G1 vs)	<0.001		<0.001	
G2	<0.001	2.740(1.901–3.951)	0.763	1.082(0.649–1.802)
G3	<0.001	5.666(3.936–8.155)	0.302	1.328(0.775–2.275)
LVI (No vs Yes)	<0.001	2.886(2.611–3.191)	<0.001	1.541(1.371–1.731)
T Stage (1 vs)	<0.001		<0.001	
2	<0.001	2.376(2.144–2.633)	<0.001	1.668(1.468–1.894)
3	<0.001	5.206(4.455–6.084)	<0.001	3.083(2.551–3.725)
4	<0.001	13.026(10.650–15.930)	<0.001	5.386(4.220–6.873)
Is	<0.001	0.411(0.309–0.548)	0.006	0.340(0.158–0.729)
0	0.591	1.712(0.241–12.173)	0.929	0.004(0.000–1.823E+050)
N Stage (0 vs)	<0.001		<0.001	
1	<0.001	2.550(2.294–2.835)	<0.001	1.935(1.676–2.234)
2	<0.001	4.161(3.590–4.823)	<0.001	2.483(2.060–2.993)
3	<0.001	8.423(7.356–9.646)	<0.001	4.278(3.572–5.123)
HR (No vs Yes)	<0.001	0.419(0.376–0.467)	<0.001	0.660(0.533–0.817)
HER2 (No vs Yes)	<0.001	1.272(1.152–1.405)	0.001	0.801(0.700–0.917)
Hormonal therapy (No vs Yes)	<0.001	0.585(0.534–0.641)	0.029	0.790(0.640–0.976)
Chemotherapy (No vs Yes)	<0.001	2.675(2.392–2.992)	<0.001	0.712(0.600–0.846)
HER2 Targeted therapy (No vs Yes)	<0.001	1.377(1.173–1.618)	0.614	0.947(0.765–1.172)
DFS				
Year of surgery (2000–2007 vs 2008–2013)	<0.001	0.778(0.715–0.846)	0.013	0.875(0.788–0.972)
Age (<50 vs ≥50)	<0.001	0.811(0.746–0.882)	<0.001	0.805(0.733–0.884)
Histologic grade (G1 vs)	<0.001		0.029	
G2	<0.001	2.747(2.044–3.691)	0.008	1.775(1.159–2.719)
G3	<0.001	4.643(3.456–6.237)	0.003	2.010(1.272–3.176)
Nuclear grade (G1 vs)	<0.001		0.009	
G2	<0.001	2.708(2.008–3.651)	0.496	1.165(0.751–1.805)
G3	<0.001	4.746(3.521–6.398)	0.248	1.317(0.825–2.101)
LVI (No vs Yes)	<0.001	2.791(2.555–3.050)	<0.001	1.716(1.547–1.902)
T Stage (1 vs)	<0.001		<0.001	
2	<0.001	2.204(2.014–2.412)	<0.001	1.622(1.453–1.812)
3	<0.001	4.288(3.711–4.954)	<0.001	2.533(2.131–3.011)
4	<0.001	9.536(7.800–11.659)	<0.001	4.530(3.557–5.768)
Is	<0.001	0.666(0.546–0.813)	0.104	0.675(0.420–1.084)
0	0.862	0.001(0.000–1.589E+32)	0.900	0.003(0.000–2.134E+036)
N Stage (0 vs)	<0.001		<0.001	
1	<0.001	2.030(1.849–2.229)	<0.001	1.424(1.256–1.613)
2	<0.001	3.236(2.826–3.705)	<0.001	1.842(1.559–2.177)
3	<0.001	6.405(5.653–7.258)	<0.001	3.167(2.694–3.723)
HR (No vs Yes)	<0.001	0.585(0.538–0.635)	0.087	0.840(0.688–1.025)
HER2 (No vs Yes)	<0.001	1.228(1.123–1.344)	<0.001	0.799(0.704–0.906)
Hormonal therapy (No vs Yes)	<0.001	0.660(0.608–0.717)	0.001	0.715(0.586–0.871)
Chemotherapy (No vs Yes)	<0.001	2.314(2.104–2.544)	0.001	0.771(0.665–0.894)
HER2 Targeted therapy (No vs Yes)	<0.001	1.620(1.426–1.841)	0.034	1.212(1.014–1.448)

CI: confidence interval; OS: overall survival; LVI: lymphovascular invasion status HR: hormone receptor; HER2: human epidermal growth factor receptor 2 DFS: disease-free survival; HER2: human epidermal growth factor receptor 2.

### Subgroup analysis by breast cancer subtype

To evaluate the factors that had an influence on chronological improvement in breast cancer survival, we performed subgroup analysis by breast cancer subtype. A total of 298 patients in P1 and 257 patients in P2 with unknown subtypes were excluded in this subgroup analysis. Table 3 shows the clinicopathological features of patients classified by subtypes in each period. The table shows trends of adjuvant treatment changing as time passed. In P1, more than 70% of patients with HR(+) had SERM as hormonal therapy. However, in P2, the proportion of patients treated with only SERM reduced and the percentage of patients treated with AI had a three-fold increase. In addition, in P2, more than half of the patients with HER2(+) subtypes received the HER2-targeted therapy, whereas only few patients were treated with HER2-targeted therapy in P1.

**Table 3:** Clinicopathological features of patients categorized by subtypes in each period.

	2000–2007	6,768				2008–2013	10,453			
	Luminal A	Luminal B	HER2	Triple negative	p-value	Luminal A	Luminal B	HER2	Triple negative	p-value
	3,633	930	941	1,264		6,366	1,031	1,356	1,700	
	No.(%)	No.(%)	No.(%)	No.(%)		No.(%)	No.(%)	No.(%)	No.(%)	
Age at diagnosis (y)					<0.001					<0.001
<50	2,397 (66.0)	651 (70.0)	486 (51.6)	780 (61.7)		3,701 (58.1)	606 (58.8)	530 (39.1)	908 (53.4)	
≥50	1,236 (34.0)	279 (30.0)	455 (48.4)	484 (38.3)		2,665 (41.9)	425 (41.2)	826 (60.9)	792 (46.6)	
T stage					<0.001					<0.001
Tis	338 (9.3)	84 (9.0)	67 (7.1)	27 (2.1)		820 (12.9)	106 (10.3)	111 (8.2)	64 (3.8)	
T1	1,991 (54.8)	434 (46.7)	442 (47.0)	571 (45.2)		3,512 (55.2)	450 (43.6)	694 (51.2)	735 (43.2)	
T2	1,167 (32.1)	348 (37.4)	369 (39.2)	582 (46.0)		1,749 (27.5)	377 (36.6)	446 (32.9)	764 (44.9)	
T3	111 (3.0)	47 (5.1)	33 (3.5)	60 (4.7)		243 (3.8)	67 (6.5)	76 (5.6)	92 (5.4)	
T4	25 (0.7)	17 (1.8)	30 (3.2)	24 (1.9)		29 (0.5)	23 (2.2)	19 (1.4)	30 (1.8)	
T0	1 (0.0)	0 (0.0)	0 (0.0)	0 (0.0)		2 (0.0)	1 (0.1)	3 (0.2)	3 (0.2)	
Unknown	-	-	-	-		13 (0.2)	7 (0.7)	7 (0.5)	12 (0.7)	
N stage					<0.001					<0.001
N0	2,336 (64.3)	547 (58.8)	586 (62.3)	780 (61.7)		4,407 (69.2)	621 (60.2)	892 (65.8)	1,115 (65.6)	
N1	939 (25.8)	279 (30.0)	219 (23.3)	308 (24.4)		1,478 (23.1)	280 (27.2)	279 (20.6)	390 (22.9)	
N2	229 (6.3)	62 (6.7)	68 (7.2)	99 (7.8)		286 (4.5)	61 (5.9)	100 (7.4)	79 (4.6)	
N3	129 (3.6)	42 (4.5)	68 (7.2)	77 (6.1)		181 (2.8)	61 (5.9)	78 (5.8)	104 (6.1)	
Unknown	-	-	-	-		14 (0.2)	8 (0.8)	7 (0.5)	12 (0.7)	
Histologic grade					<0.001					<0.001
G1	330 (9.1)	42 (4.5)	12 (1.3)	13 (1.0)		541 (8.5)	12 (1.2)	3 (0.2)	14 (0.8)	
G2	2,075 (57.1)	443 (47.6)	277 (29.4)	344 (27.2)		4,111 (64.6)	488 (47.3)	350 (25.8)	367 (21.6)	
G3	610 (16.8)	283 (30.4)	489 (52.0)	801 (63.4)		791 (12.4)	402 (39.0)	846 (62.4)	1,212 (71.3)	
Unknown	618 (17.0)	162 (17.4)	163 (17.3)	106 (8.4)		923 (14.5)	129 (12.5)	157 (11.6)	107(6.3)	
Nuclear grade					<0.001					<0.001
G1	304 (8.4)	31 (3.3)	3 (0.3)	10 (0.8)		673 (10.6)	14 (1.4)	4 (0.3)	16 (0.9)	
G2	2,022 (55.7)	404 (43.4)	244 (25.9)	309 (24.4)		4,636 (72.8)	541 (52.5)	379 (27.9)	380 (22.4)	
G3	561 (15.4)	260 (28.0)	485 (51.5)	728 (57.6)		877 (13.8)	458 (44.4)	935 (69.0)	1,265 (74.4)	
Unknown	746 (20.5)	235 (25.3)	209 (22.2)	217 (17.2)		180 (2.8)	18 (1.7)	38 (2.8)	39 (2.3)	
Lymphovascular invasion					<0.001					<0.001
Negative	2,150 (59.2)	469 (50.4)	523 (55.6)	755 (59.7)		4,251 (66.8)	617 (59.8)	926 (68.3)	1,235 (72.6)	
Positive	704 (19.4)	204 (21.9)	210 (22.3)	296 (23.4)		1.220 (19.2)	303 (29.4)	267 (19.7)	330 (19.4)	
Unknown	779 (21.4)	257 (27.6)	208 (22.1)	213 (16.9)		895 (14.1)	111 (10.8)	163 (12.0)	135 (7.9)	
Hormonal therapy					<0.001					<0.001
No	215 (5.9)	63 (6.8)	796 (84.6)	1,078 (85.3)		264 (4.1)	63 (6.1)	1,230 (90.7)	1,577 (92.8)	
AI	268 (7.4)	67 (7.2)	12 (1.3)	18 (1.4)		1,470 (23.1)	237 (23.0)	29 (2.1)	30 (1.8)	
SERM	2,657 (73.1)	705 (75.8)	131 (13.9)	167 (13.2)		3,215 (50.5)	640 (62.1)	94 (6.9)	87 (5.1)	
SERM+LHRH analog	452 (12.4)	67 (7.2)	2 (0.2)	1 (0.1)		1,375 (21.6)	82 (8.0)	2 (0.1)	6 (0.4)	
Unknown	41 (1.1)	28 (3.0)	0 (0.0)	0 (0.0)		42 (0.7)	9 (0.9)	1 (0.1)	0 (0.0)	
Neoadjuvant therapy					0.451					<0.001
Yes	3 (0.1)	0 (0.0)	0 (0.0)	2 (0.2)		505 (7.9)	181 (17.6)	197 (14.5)	271 (15.9)	
No	3,630 (99.9)	930 (100.0)	941 (100.0)	1,262 (99.8)		5,861 (92.1)	850 (82.4)	1,156 (85.5)	271 (15.9)	
Chemotherapy					<0.001					<0.001
No	1,722 (47.4)	329 (35.4)	133 (14.1)	83 (6.6)		3,707 (58.2)	335 (32.5)	419 (30.9)	216 (12.7)	
Anthracyclin-based	945 (26.0)	305 (32.8)	369 (39.2)	669 (52.9)		839 (13.2)	279 (27.1)	423 (31.2)	799 (47.0)	
Anthracyclin- and taxaned-based	666 (18.3)	205 (22.0)	250 (26.6)	297 (23.5)		1,283 (20.2)	360 (34.9)	399 (29.4)	371 (21.8)	
CMF	123 (3.4)	27 (2.9)	24 (2.6)	55 (4.4)		5 (0.1)	4 (0.4)	0 (0.0)	7 (0.4)	
Others	11 (0.3)	1 (0.1)	97 (10.3)	49 (3.9)		12 (0.2)	14 (1.4)	50 (3.7)	20 (1.2)	
Unknown	166 (4.6)	63 (6.8)	68 (7.2)	111 (8.8)		520 (8.2)	39 (3.8)	65 (4.8)	287 (16.9)	
HER2-targeted therapy					<0.001					<0.001
Yes	4 (0.1)	9 (1.0)	16 (1.7)	5 (0.4)		60 (0.9)	617 (59.8)	757 (55.8)	26 (1.5)	
No	3,629 (99.9)	921 (99.0)	925 (98.3)	1,259 (99.6)		6,306 (99.1)	414 (40.2)	599 (44.2)	1,674 (98.5)	

Luminal A: HR(+)/HER2(–); Luminal B: HR(+)/HER2(–); HER2: HR(–)/HER2(–); Triple negative: HR(–)/HER-2(–); HR: hormone receptor; HR(+): estrogen receptor (+) and/or progesterone receptor (+); HER2: human epidermal growth factor receptor 2; Stage: initial clinical stage for patients who underwent neoadjuvant therapy and pathological stage for patients who did not; AI: aromatase inhibitor; SERM: selective estrogen receptor modulator; LHRH: luteinizing hormone-releasing hormone; CMF: cyclophosphamide, methotrexate, and 5-fluorouracil.

**Fig 2:**
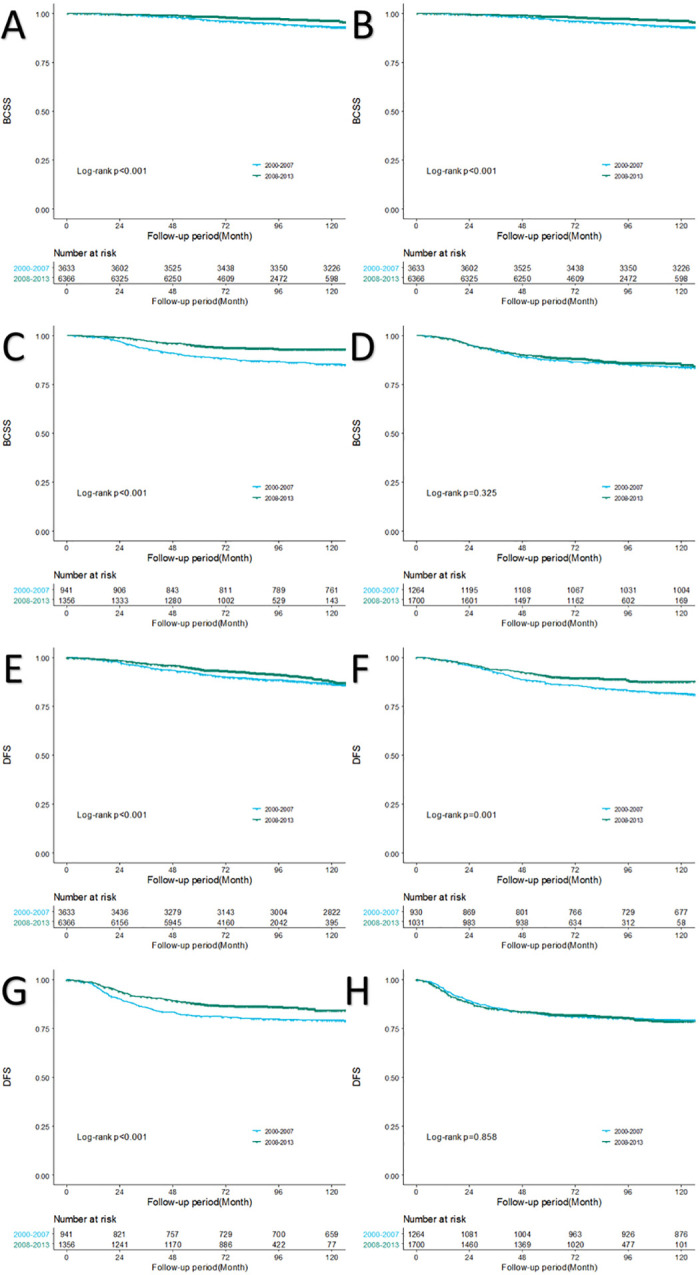
Chronological changes of breast cancer-specific survival (BCSS) and disease-free survival (DFS) in Korean breast cancer patients; subgroup analysis by subtype. (A) BCSS of subtype HR(+)/HER-2(–). (B) BCSS of subtype HR(+)/HER2(+). (C) BCSS of subtype HR(–)/HER-2(+). (D) BCSS of subtype HR(–)/HER2(–). (E) DFS of subtype HR(+)/HER2(–). (F) DFS of subtype HR(+)/HER2(+). (G) DFS of subtype HR(–)/HER2(+). (H) DFS of subtype HR(–)/HER2(–). HR: hormone receptor; HER2: human epidermal growth factor receptor 2.

When we performed the Kaplan–Meier survival analysis according to the time of surgery in each subtype, both the BCSS and DFS of the P2 cohort were significantly better than those of P1 cohort in every subtype except for the triple negative subtype, with a p-value of 0.325 and 0.858, respectively (**Fig 2**). Subtypes with HER2 overexpression showed more dramatic improvement; in luminal B subtypes, there was a 5-year BCSS increase from 94.7% in P1 to 97.3% in P2 and a 5-year DFS increased from 86.7% in P1 to 90.5% in P2. In HER2 subtypes the 5-year BCSS increased from 89.2% in P1 to 95.1% in P2, and the 5-year DFS increased from 81.5% in P1 to 87.7% in P2.

When we performed an Kaplan–Meier survival analysis on DMFS by subtype, improvemet in P2 to a significant degree in every subtype except for triple negative breast cancer (**S1 Fig**).

**Fig 3:**
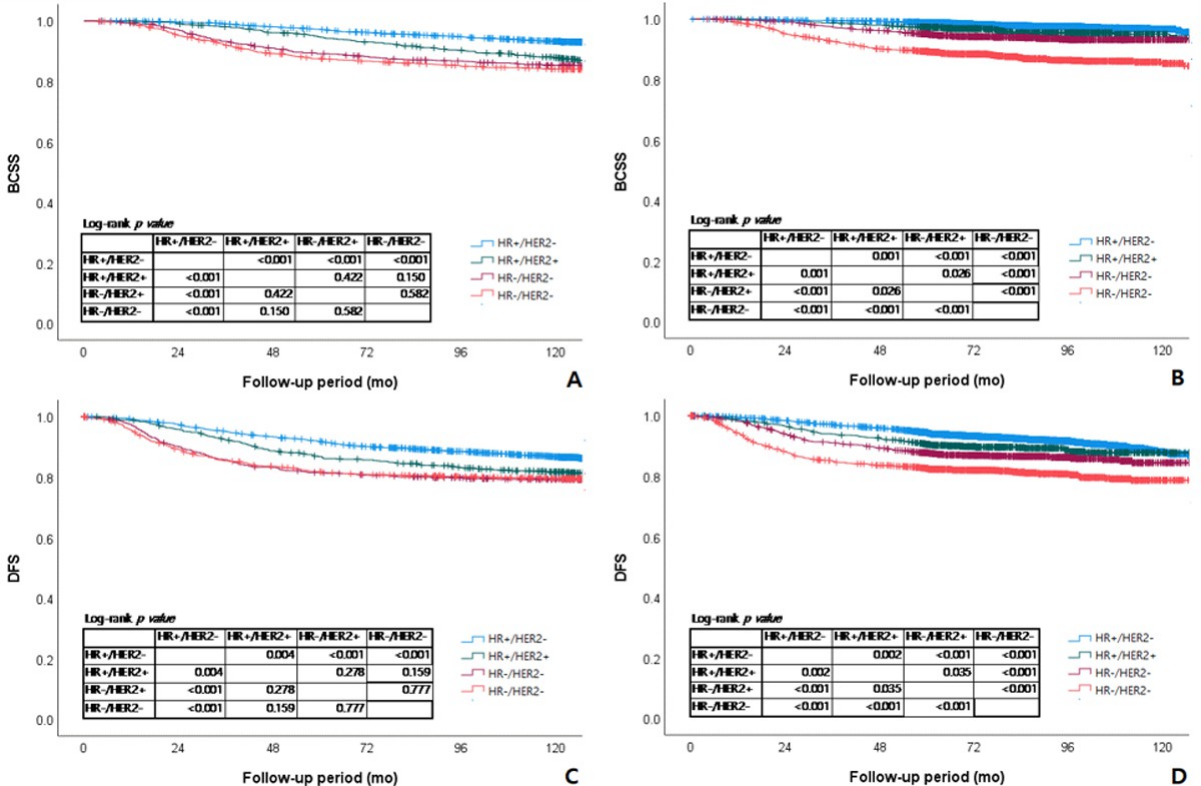
Breast cancer-specific survival (BCSS) and disease-free survival (DFS) in Korean breast cancer patients according to the subtype in each period. (A) BCSS of patients who had surgery between 2000–2007. (B) BCSS of patients who had surgery between 2008–2013. (C) DFS of patients who had surgery between 2000–2007. (D) DFS of patients who had surgery between 2007–2013. HR: hormone receptor; HR(+): estrogen receptor (+) and/or progesterone receptor (+); HER2: human epidermal growth factor receptor 2.

We performed a Kaplan–Meier survival analysis according to the subtype to compare the trend of survival differences between subtypes in each period. In P1, those with the luminal A subtype had significantly better BCSS and DFS than those with the luminal B, HER2, or triple negative subtype(luminal A vs. luminal B: BCSS, p<0.001; DFS, p = 0.004). The comparison between the luminal B, HER2, and triple negative subtypes did not show any significant difference in BCSS and DFS (**Fig 3A and 3C**). In P2, as in P1, luminal A subtype yielded significantly better BCSS and DFS than luminal B, HER2, and triple negative subtypes, with luminal A versus luminal B p-value = 0.001 and 0.002, respectively. Unlike P1, in P2, luminal B and HER2 subtypes showed survival curves closer to the survival curve of the luminal A subtype and both luminal B and Her2 subtypes showed significantly better survival than the triple negative subtype in both BCSS and DFS (**Fig 3B and 3D**).

## Discussion

In our previous study, we analyzed the chronological survival trend in breast cancer patients diagnosed at Asan Medical Center from 1993 to 2008 by dividing them into three periods (P1, 1993–1997; P2,1998–2002; P3, 2003–2008) and found a significant improvement in the 5-year BCSS rate from 82.8% in P1 to 92.6% in P3 (p<0.001). In the subgroup analysis by stage, the survival improvement was especially apparent in stage III breast cancer (5-year BCSS: P1, 57.4% vs. P3, 80.0%; p<0.001), indicating that advancements in adjuvant therapy may have influenced the chronological survival improvement [4]. Since that study, we have collected and updated the Asan data, and in the current study, we evaluated the changing patterns of survival and recurrence of Korean breast cancer patients over a 14-year period (2000–2013).

Unlike previous studies that used data from population registries, this was as single-center study. Although single center study can have potential risk for selection bias, there are some advantages. For example, the enrolled patients in this study had a relatively uniform diagnosis and treatment environment considering the duration of data collected.

Our current study showed improvements in OS, BCSS and DFS over time (**Fig 1**). The 5-year BCSS was 96.6% during the latter period (2008–2013), which was a significant improvement from 94.0% in P1 (p<0.001). Our results were similar to those of other studies that were based on larger populations; for American patients with breast cancer diagnosed during 2003–2009 that were registered in the Surveillance, Epidemiology, and End Results database, the age-standardized 5-year relative survival rate was 89.2% [16], while the 5-year relative survival rate of patients with breast cancer diagnosed in 2006–2010 in the Korea central registration statistics was 91.2% [17].

Many other studies worldwide have suggested early detection and an increased proportion of less aggressive types of breast cancer as possible explanations for the improvement in survival rate of patients with breast cancer [9, 10]. In our study, a similar pattern was observed. More patients were diagnosed at Tis stage or N0 stage in P2 compared with the earlier period (Table 1). We expected that the nationwide screening program for breast cancer would lead to earlier detection and would influence improvement in survival. In addition, the proportion of the luminal A subtype increased from 51.4% in P1 to 59.4% in P2, whereas other subtypes decreased (Table 1). As luminal A breast cancer yielded the highest BCSS among the other subtypes (**Fig 3A and 3B**), an increased proportion of luminal A cancer likely had an influence on improvement in chronological survival.

In our study, DFS also showed improvement over time, with 87.9% in P1 to 91.2% in P2. When we performed analysis on DMFS, improvement was seen to significant degree, with the 5-year DMFS 90.7% in P1 and 94.1% in P2 (**Fig 1D**). In the subgroup analysis by breast cancer subtypes, significantly better DFS and DMFS were shown in luminal A, luminal B, and HER2 subtypes in the later period (**Figs 2 and S1**), and the difference was more dramatic in subtypes with HER2(+). When we compared BCSS and DFS by subtype in each period, survivals of those with the HER2 and triple negative subtypes did not show a significant difference in the earlier period, but in the later period, the HER2 subtype had better survival than the triple negative subtype by a significant degree (**Fig 3**). Improvement in DMFS can likely be attributed to advancement in systemic therapy. As survival differences were shown in luminal A, luminal B, and HER2 subtypes, we investigated the types of adjuvant therapy used in each subtype (Table 3). In cases of hormonal therapy, the mainly administered treatment in the earlier period was SERM, though the use of AI and LHRH analogs increased in the later period. For example, the proportion of luminal A subtype patients treated with AI was 7.4% in P1 and 23.1% in P2. It has been reported that postmenopausal patients treated with AI have better survival than those treated with SERM [18]. Therefore, advancement in hormonal therapy can be considered as one of the factors that influenced chronological survival improvement. Use of trastuzumab in the adjuvant or neoadjuvant setting for breast cancer was not covered by the Korean National Health Insurance until 2007. Therefore, most patients with the HER2(+) subtype did not receive target therapy in P1 (Table 3). In 2007, HER2(+) patients with positive lymph nodes were covered by insurance, and by 2010, the insurance cover extended to patients with primary cancer size ≥1 cm. In our data, more than half of the patients with HER2(+) subtypes received target therapy in P2 (Table 3), which means most of the patients allowed by insurance were treated with the target therapy. It is a well-known fact that trastuzumab improved survival of HER2(+) breast cancer patients [13, 19]. Use of target therapy can also be considered as one of the factors that influenced the chronological survival improvement.

In addition, the time factor itself was an independent and significant factor associated with improved OS and DFS (Table 2). There was a 22.7% improvement in OS between both periods. This increase was most likely a surrogate for improvements in detection, better preoperative diagnostic planning, better multidisciplinary decision making, and a thorough pathological investigation. The overall gains from the time effect were most likely due to a combination of other technological, economic, and social factors [4].

Our present study had a number of limitations. First, as in all single-institution, retrospective, observational studies, there was a potential for selection bias. Second, the differences in the follow-up period between the investigated periods might be a limitation; the median follow-up was 155.9 months for P1 and 85.9 months for P2. Third, because we did not exclude neoadjuvant patients, our results might have been affected.

In conclusion, this large-scale single-centered study with more than 10,000 patients revealed improvement in survival of breast cancer over a 14-year period. As the analysis showed a chronological recurrence rate difference in DMFS, we concluded that the recent improvement in Korean breast cancer patient outcomes might be due to advancements in systemic treatment. Moreover, as the time factor itself was a significant factor for survival improvement, better technological, economic, and social statuses might also be one of reasons for survival improvement.

## Supporting information

S1 Fig Chronological changes of distant metastasis-free survival (DMFS) in Korean breast cancer patients according to the subtype. (A) HR+/HER2–. (B) HR+/HER2+. (C) HR–/HER2+. (D) HR–/HER2–. HR: hormone receptor; HER2: human epidermal growth factor receptor 2.
(TIF)
