# Supplementary figures and images for: Improvement of survival in Korean breast cancer patients over a 14-year period: A large-scale single-center study
Source: PLoS One. 2022 Mar 16;17(3):e0265533. doi: 10.1371/journal.pone.0265533 (PMC8926243; doi:10.1371/journal.pone.0265533)

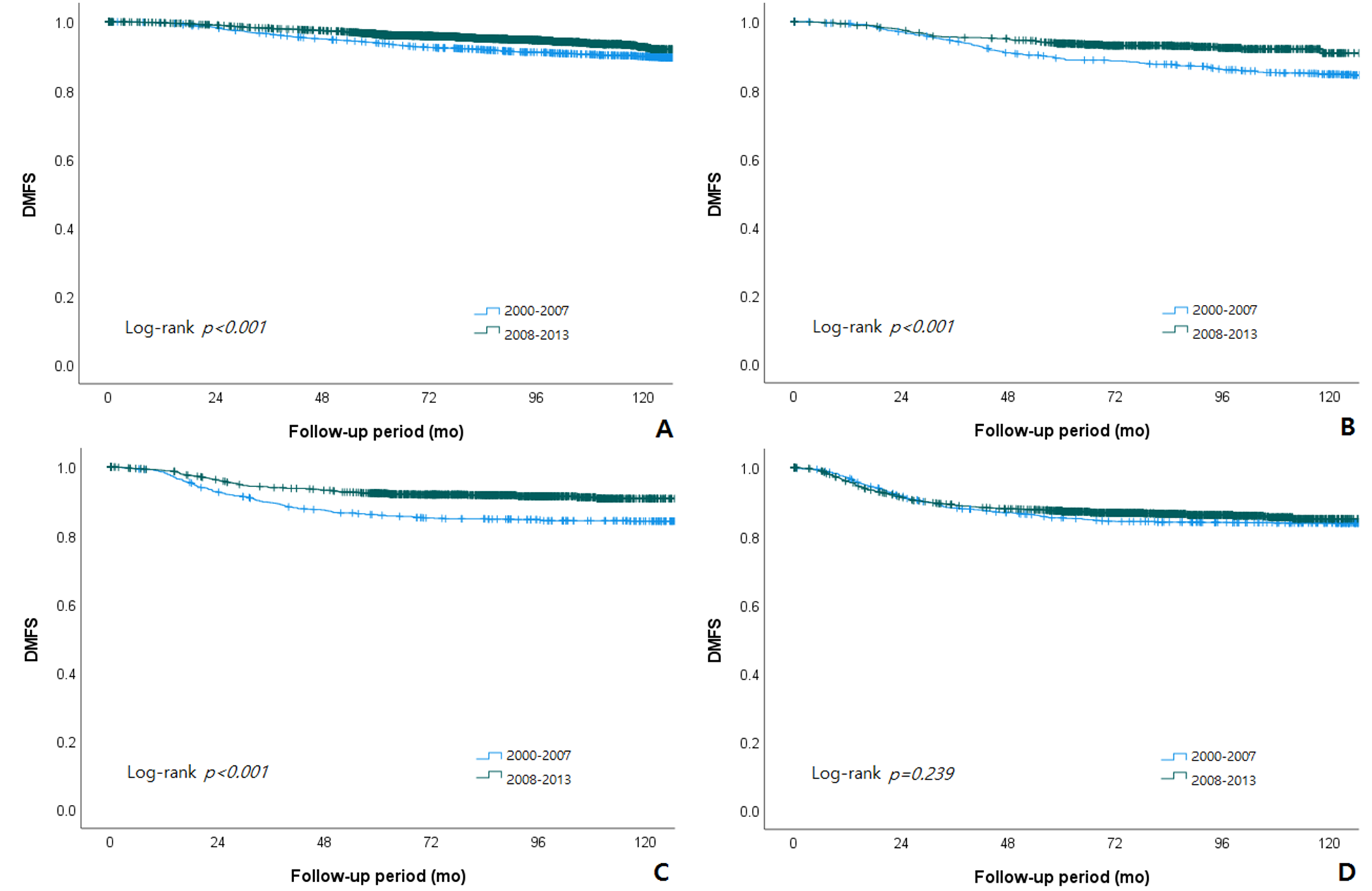

Supplement: S1 Fig — (A) HR+/HER2–. (B) HR+/HER2+. (C) HR–/HER2+. (D) HR–/HER2–. HR: hormone receptor; HER2: human epidermal growth factor receptor 2. (TIF) [file pone.0265533.s001.tif]
